# Mitochondrial DNA Content May Not Be a Reliable Screening Biomarker for Live Birth After Single Euploid Blastocyst Transfer

**DOI:** 10.3389/fendo.2021.762976

**Published:** 2021-11-16

**Authors:** Xuanyou Zhou, Xueli Liu, Weihui Shi, Mujin Ye, Songchang Chen, Chenming Xu

**Affiliations:** ^1^ International Peace Maternity and Child Health Hospital, School of Medicine, Shanghai Jiao Tong University, Shanghai, China; ^2^ Shanghai Key Laboratory of Embryo Original Diseases, Shanghai, China; ^3^ Obstetrics and Gynecology Hospital, Fudan University, Shanghai, China

**Keywords:** mitochondrial DNA, live birth, IVF, embryo viability, quiet embryo hypothesis

## Abstract

An increasing number of studies have related the mitochondrial DNA (mtDNA) content to embryo viability and transfer outcomes. However, previous studies have focused more on the relationship between mtDNA and embryo implantation, few studies have studied the effect of the mtDNA content on live birth. In the study, we investigated whether mtDNA content is a reliable screening biomarker for live birth after single blastocyst transfer. A total of 233 couples with 316 blastocyst stage embryos undergoing *in vitro* fertilization treatment and pre-implantation genetic testing analysis were included in the study. All embryos were chromosomally normal and had undergone single-embryo transfers. There was no significant difference observed in the blastocyst mtDNA content among the live birth, miscarriage and non-implanted groups (p=0.999), and the mtDNA content in blastocysts from the miscarriage and live birth groups was similar [median (interquartile range), 1.00*10^8^(7.59*10^7^- 1.39*10^8^) *vs* 1.01*10^8^ (7.37*10^7^- 1.32*10^8^)]. Similarly, no significant association was observed between mtDNA content and embryo implantation potential (p=0.965). After adjusting for multiple confounders in a logistic regression analysis with generalized estimating equations, no associations between mtDNA content and live birth were observed in all blastocysts, Day-5 and Day-6 blastocysts (p=0.567, p=0.673, p=0.165, respectively). The live birth rate was not significantly different between blastocysts with an elevated mtDNA content and blastocysts with a normal mtDNA content (26.7% *vs* 33.6% p=0.780). Additionally, there was no linear correlation between the mtDNA content and maternal age (p=0.570). In conclusion, the mtDNA content does not seem to be a potential biomarker for embryo transfer outcomes (i.e., implantation and live birth) based on the existing testing tools. Embryos with an elevated mtDNA content also have development potential for successful live birth.

## Introduction

Infertility affects almost 15% of couples worldwide who try to conceive (1, 2), and approximately 30% of these couples cannot explain the cause of their infertility (3). Assisted reproductive technology (ART), including *in vitro* fertilization (IVF) and intracytoplasmic sperm injection (ICSI) has become the most effective treatment for infertility in both developed and developing countries (4, 5). However, improving IVF success rate is still a challenging issue in clinical IVF practice. Despite numerous technical advances in IVF technology, the overall IVF success rate (measured as a live birth)remains low at 16.6-30% (6, 7).

In the past, the success of IVF depended highly on the number of transferred embryos. Increasing the number of transferred embryos may lead to an increased rate of multiple pregnancy, which is one of the most common and serious complications of ART and increases the likelihood of adverse obstetric and perinatal outcomes (8). Currently, the decision of the best embryo for transfer is made based on morphological parameters observed during embryonic development in the IVF laboratory (9, 10). In addition, the widespread use of pre-implantation genetic testing (PGT) has improved the rate of successful pregnancy and reduced the risk of miscarriage (11–13). However, even if the transferred embryo has normal morphological and chromosomal results, some embryos still fail to implant, or the embryos are successfully implanted but fail to result in a live birth (14, 15).

Mitochondria are highly dynamic organelles that play essential roles in the regulation of reproductive processes (16, 17) and intracellular powerhouses that generate cellular energy (adenosine triphosphate, ATP) through the oxidative phosphorylation (OXPHOS) system (18). Human cells harbor hundreds to thousands of mitochondria, each of which contains 2–10 copies of mtDNA in its matrix (19), while in oocytes, there is only one copy per cell (20, 21). Mature oocytes contain more mtDNA than other cells to meet energy demands in the process of fertilization and the early stages of embryonic development (22, 23). Many animal studies have shown that mitochondrial function affects reproductive outcomes (24–26). Recently, an increasing number of studies have related higher mtDNA content in embryos to poor implantation potential and suggested mtDNA content to be a novel tool to predict embryo viability (27–30). Fragouli and Ravichandran et al. established a screening threshold which embryos with mtDNA content above the threshold was less likely to implant (29, 30). Whereas subsequent studies have reached conflicting conclusions (31–33). These contradictory findings limit the application of mtDNA in the accurate assessment of embryo viability in clinical practice. Besides, the most commonly used technology for mtDNA quantification is to calculate the ratio of mtDNA to nuclear DNA (nDNA) in samples using next-generation sequencing (NGS) based or quantitative polymerase chain reaction (qPCR) based methods. In fact, the content of nuclear genomes is not equal across embryos, therefore using solely nuclear genomes for normalization is not enough. To correct for this bias, Victor et al. first proposed the correction factor of embryo’s sex and ploidy for the evaluation of mtDNA content for both NGS-based and qPCR-based technologies and they found no association between mtDNA and implantation potential (34), similar conclusions were later confirmed by another study used the correction factor (35). In addition, El-Damen et al. investigated whether mtDNA content is associated with miscarriage rate and reported that the mtDNA content could not predict the miscarriage of blastocysts after transfer (36).

In general, previous studies have focused more on the relationship between mtDNA and embryo implantation, while few studies have studied the effect of the mtDNA content on live birth. In fact, implantation rate, clinical pregnancy and live birth are commonly used to measure the success of ART treatments. However, for couples who desire a baby, live birth is the only indicator of successful IVF treatment. In addition, patient exclusion criteria have been neglected in some prior studies, which might affect the credibility of the existing results. This study aims to investigate the potential relationship between the mtDNA content and live birth after implantation.

## Materials

### Study Design

This retrospective study investigated the mtDNA content in 316 blastocyst-stage embryos produced by 233 patients undergoing IVF treatment and PGT analysis at the Reproductive Genetic Centre of International Peace Maternal and Child Health Hospital (IPMCH) of Shanghai Jiao Tong University School of Medicine during the period from 2016 to 2020. Next-generation sequencing (NGS)-based PGT was performed for the analysis of all blastocyst biopsies on day 5 or 6. Each embryo included in the study was chromosomally normal and had undergone single embryo transfer (SET) to the uterus. The inclusion criteria were as follows: (1) female patients between the ages of 20 to 45 years undergoing IVF and PGT due to various indications with the evaluation of mtDNA content; (2) patients with normal chromosomal and copy number variation (CNV) results; and (3) patients who had undergone SET. The exclusion criteria were as follows: (1) patients who were diagnosed with endocrine/metabolic disease or were under medication treatment; (2) patients with autoimmune disease and (3) patients with endometrial disease (e.g., endometrial hyperplasia, submucous myoma or malignancy, as determined by pathological examination).

The primary objective of this study was to evaluate the association between the mtDNA content and live birth, the secondary objective was to assess the relationship between the mtDNA content and maternal age. The study was approved by the ethics committee of the IPMCH. All patients were informed of details of the procedure and signed an informed-consent agreement.

### Oocyte Retrieval and Embryo Culture

All patients underwent treatment with the standard ovarian stimulation protocols with exogenous gonadotrophins to promote multi-follicular development according to the standardized IVF procedure at our hospital. After ovarian stimulation, human chorionic gonadotropin (hCG, 5000–10,000 IU, Livzon Pharmaceutical Group Co, Ltd, China) was injected subcutaneously to induce the final maturation of follicles when the dominant follicle diameter was 16–18 mm, and oocyte retrieval was performed 36–38 h later by transvaginal ultrasound-guided needle aspiration. All oocytes were fertilized using ICSI and then transferred to embryo culture medium. Embryos were cultured in a tri-gas incubator (5% O_2_, 6% CO_2_, and 89% N_2_ at 37°C) for the first 5–6 days, and the formed blastocysts then underwent trophectoderm (TE) biopsy.

### Trophectoderm Biopsy

Trophectoderm cell biopsy was performed on expanding and hatched blastocysts. On day 5 or 6 after ICSI, TE cells were subjected to biopsy, 5 to 10 TE cells were aspirated with the biopsy needles, followed by laser-assisted removal, The TE cells were rinsed 3 times with phosphate-buffered saline (Vitrolife, Sweden) and were rapidly transferred to a 0.2 ml PCR tube for DNA amplification. After biopsy, the blastocysts were transferred to the culture medium.

Assessment of embryo morphology was performed based on the grading system described by Gardner and Schoolcraft (37). Good blastocysts included grades 6, 5, 4, 3AA and 6, 5, 4, 3AB, and 3BA. Average blastocysts included grades 6, 5, 4, and 3BB while poor blastocysts included all expansion stages with TE or ICM (inner cell mass) grade C, as described by El-Damen et al. (36).

### Embryo Cryopreservation and Frozen/Thawed Embryo Transfer

All blastocysts were transferred in frozen-thawed embryo transfer (FET) cycles. Embryo vitrification and warming procedures were achieved using a commercial Kitazato Vitrification kit (Kitazato, Tokyo, Japan) and warming Media (Kitazato, Tokyo, Japan) according to the Cryotop^®^ method described by Kuwayama et al. (38). Endometrial preparation for frozen-thawed cycles included natural cycle and hormone replacement therapy (HRT). Luteal support was provided after embryo transfer. For natural cycle, Dydrogesterone Tablets (10mg po tid, Abbott Healthcare Products B.V.) was administered from the day of ovulation until the 12th week of pregnancy. For patients with HRT, luteal phase support was performed from the start of endometrium transformation and continued daily until 12 weeks of gestation with a routine protocol: Progesterone Sustained-release Vaginal Gel (90mg pv qd, Fleet Laboratoires Ltd), Complex Packing Estradiol Tablets/Estradiol and Dydrogesterone Tablets (one Tablet pv bid, containing 1mg Estradiol and 10mg Dydrogesterone, Abbott Healthcare Products B.V.), Dydrogesterone Tablets (10mg po tid, Abbott Healthcare Products B.V.) and Estradiol Valerate Tablets (3mg po bid, Progynova).

### DNA Amplification and Next Generation Sequence

Genomic DNA from each sample was subjected to library preparation based on the guidelines of the VeriSeq PGS kit (Illumina, Santiago, USA). The elementary procedure consists of DNA quantification, tagmentation, PCR, library normalization, pooling and loading. The final products were sequenced on a MiSeq system (Illumina, San Diego, USA).

### mtDNA Quantification *via* NGS

Sequencing reads were aligned to the hg19 assembly using the Burrows-Wheeler Aligner (BWA) with default parameters (39), followed by duplicate read removal and GC-bias correction using the Genome Analysis Toolkit (GATK) and DeepTools (40, 41). The number of mtDNA and nDNA mapped reads was counted by idxstats in Samtools (42). The final mtDNA content was calculated based on the following formula referring to Victor et al (34). Aneuploidy was detected as previously described (43).


$$\text{mtDNA content}=(\text{mtDNA}/\text{nDNA})*(\text{genome size})/(\mathrm{mtDNA size})*1000000*{\text{F}}_{\text{NGS}}$$

(F_NGS_ was used to correct for the effect of sex on the results.)

### Outcome Measures

Embryo implantation was defined as intrauterine gestational sacs visualized by transvaginal ultrasound (44); live birth was defined as the delivery of a live infant after 24 weeks gestation (45, 46); miscarriage was characterized as the loss of pregnancy before 20 completed weeks; and early miscarriage was defined as the loss of pregnancy before 12 weeks of gestation (47).

### Statistical Analysis

R (v 4.0.3) and SPSS 26.0 for Windows (IBM Corp, USA) were used for statistical analysis (48). The comparison of quantitative variables between groups were evaluated by Kruskal-Wallis Test or Mann-Whitney test using Ggstatsplot (v 0.8.0) (49). The comparison of categorical variables between groups was performed using the chi-square test and Fisher’s exact chi-square test. Linear and Logistic Regression were performed by Ggstatsplot (v 0.8.0) and stats (v 4.0.3) packages. Ggplot2 (v 3.3.3) and plotROC (v 2.2.1) packages was used for data visualization (50, 51). P values and adjusted ORs were calculated using logistic regression with generalized estimating equations.

## Results

**Figure 1:**
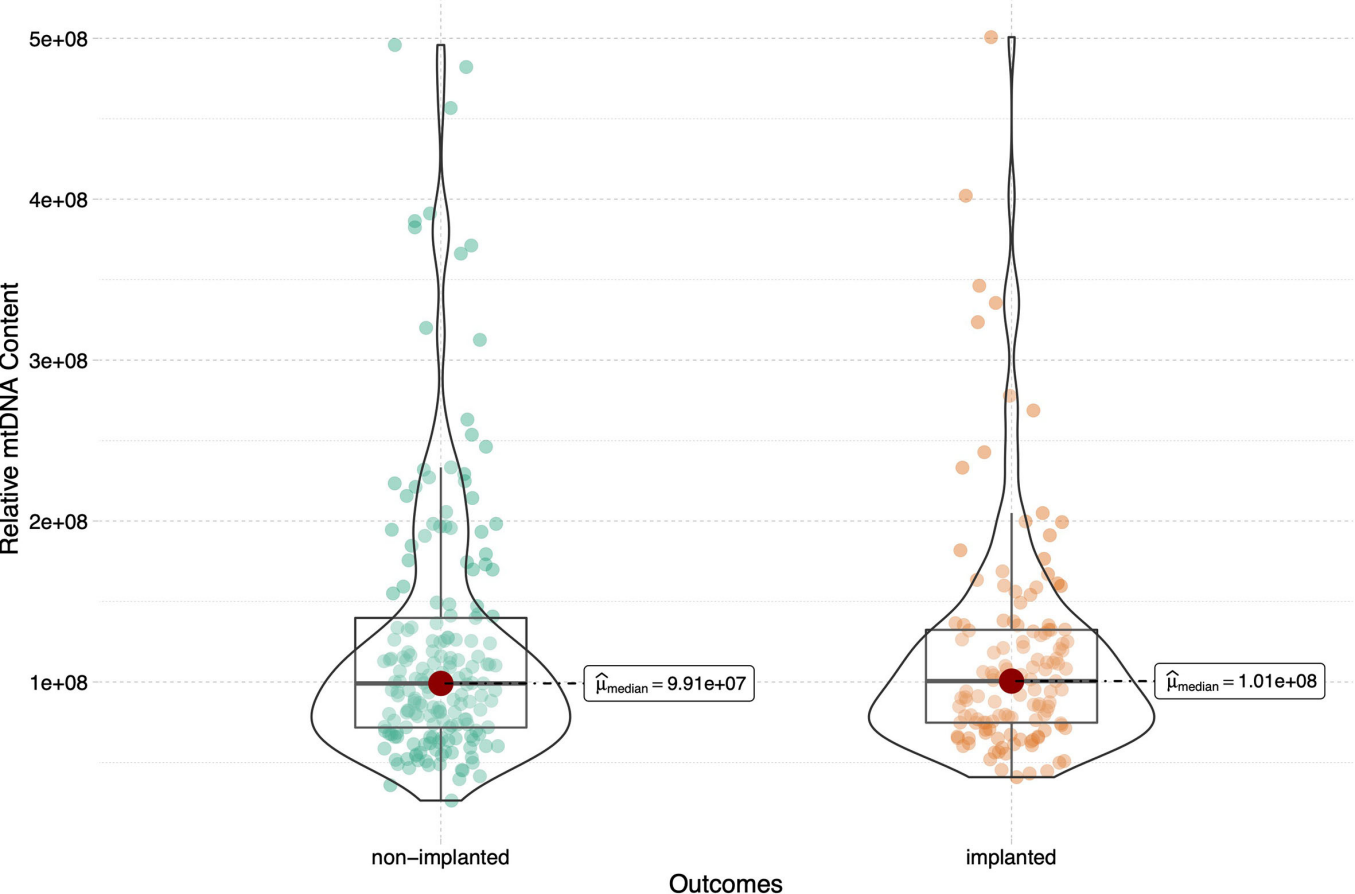
Relative mtDNA content in women between groups of embryos that did and did not implant. 316 blastocysts were transferred in the current study, 185 blastocysts failed to implant and the remaining 131 blastocysts were implanted successfully. The Mann-Whitney test was performed to compare mtDNA content between two groups (p=0.965).

In total, 316 euploid blastocysts from 233 couples were finally included in the study. The relative quantity of mtDNA in each sample was measured using NGS and corrected by a correction factor. We compared the content of mtDNA in blastocysts between different transfer outcomes. The implanted and non-implanted embryos were first analysed, and the epidemiological and clinical characteristics in these two groups were similar except for maternal age (32.1 ± 4.7 *vs* 33.2 ± 4.7, p=0.05) and biopsy day (p=0.019), data as shown in **Table 1**. The relative mtDNA content was 1.01*10^8^ (7.47*10^7^-1.32*10^8^) in the implanted group and 9.91*10^7^(7.08*10^7^-1.40*10^8^) in the non-implanted group. Unexpectedly, no significance was observed between the implanted and non-implanted groups (p=0.965, **Figure 1**).

**Table 1:** The epidemiological and clinical characteristics of the patients undergoing IVF/PGT treatment.

Characteristic	Implanted N=131	Non-implanted N=185	p value
**Maternal age, y, mean ± SD**	32.1 ± 4.7	33.2 ± 4.7	0.050
**paternal age, y, mean ± SD**	34.5 ± 5.9	34.5 ± 5.2	0.733
**BMI, kg/m2, mean ± SD**	21.4 ± 2.4	21.6 ± 2.4	0.277
**Education, n (%)**			0.911
Senior high school degree or less	19 (38.8)	30 (61.2)	
college degree	97 (41.8)	135 (58.2)	
post-graduate degree	15 (42.9)	20 (57.1)	
gravidity	1 (0-2)	0 (0-2)	0.248
parity	0 (0-0)	0 (0-0)	0.483
**Cycle protocols, n (%)**			0.896
GnRH agonist protocol	51 (41.8)	71 (58.2)	
Antagonist protocol	70 (41.9)	97 (58.1)	
microdose flare protocol	9 (40.9)	13 (59.1)	
Other protocol	1 (20.0)	4 (80.0)	
**Indications for PGT, n (%)**			0.861
chromosomal abnormalities	44 (40.7)	64 (59.3)	
Advanced age	16 (37.2)	27 (62.8)	
Male factor	19 (45.2)	23 (54.8)	
Recurrent implantation failure	6 (42.9)	8 (57.1)	
Recurrent miscarriage	21 (48.8)	22 (51.2)	
Patient request	25 (37.9)	41 (62.1)	
**Infertility type, n (%)**			0.400
Primary	47 (44.8)	58 (55.2)	
Secondary	84 (39.8)	127 (60.2)	
**bFSH, IU/L**	7.1 ± 2.2	7.3 ± 2.4	0.377
**bLH, IU/L**	5.0 ± 2.5	4.5 ± 2.1	0.164
**bEstradiol, pmol/L**	144.5 ± 75.5	140.5 ± 66.0	0.800
**gonadotropin duration, days**	10.0 ± 2.6	9.8 ± 2.3	0.741
**Gonadotropin dosage, IU**	2326.5 ± 725.6	2359.0 ± 738.0	0.626
**endometrial thickness, mm**	9.4 ± 1.5	9.4 ± 1.5	0.914
**FET type, n (%)**			0.444
Natural cycle	42 (38.5)	67 (61.5)	
HRT cycle	89 (43.0)	118 (57.0)	
**Biopsy day, n (%)**			0.019*
5	38 (53.5)	33 (46.5)	
6	93 (38.0)	152 (62.0)	
**TE quality, n (%)**			0.136
A	6 (60.0)	4 (40.0)	
B	91 (44.0)	116 (56.0)	
C	34 (34.3)	65 (65.7)	
**ICM quality, n (%)**			0.068
A	20 (55.6)	16 (44.4)	
B	111 (39.6)	169 (60.4)	
C	0 (0)	0 (0)	
**Blastocyst quality, n (%)**			0.078
Good	20 (55.6)	16 (44.4)	
Average	77 (42.5)	104 (57.5)	
Poor	34 (34.3)	65 (65.7)	

Continuous variables were calculated by Mann-Whitney test, categorical variables were calculated by chi-squared test; BMI, body mass index; PGT, preimplantation genetic testing; TE, Trophectoderm; ICM, inner cell mass; bLH, basic luteinizing hormone; bFSH, basic follicle-stimulating hormone; bAMH, basic anti-Müllerian hormone; FET, frozen-thawed embryo transfer; HRT, hormone replacement therapy; *p<0.05.

**Figure 2:**
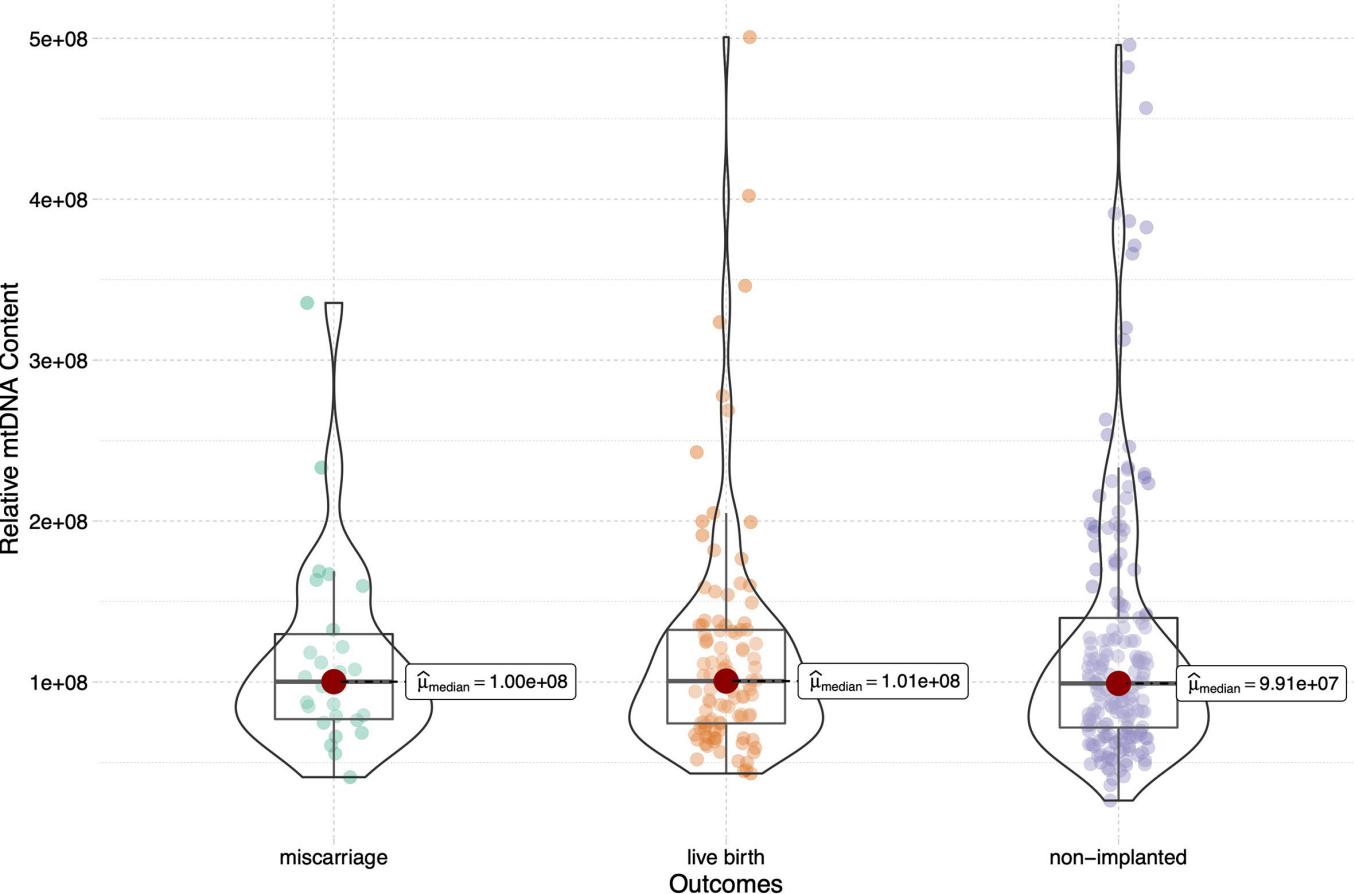
Relative mtDNA content stratified by embryo transfer outcomes (live birth, miscarriage and non-implanted) for the 316 transferred blastocysts. Kruskal-Wallis test was used to compare the mtDNA content among groups (p=0.999).

To assess the relationship between the mtDNA content and live birth after embryo implantation, we then sub-divided the implanted group into the live birth and miscarriage groups, and compared the mtDNA content among the live birth, miscarriage and non-implanted groups, as shown in **Figure 2**. The epidemiological and clinical characteristic data are summarized in **Table 2**. There was no significance in the mtDNA content among groups (p=0.999, **Figure 2**), blastocysts in miscarriage group did not show a higher mtDNA level than those in the live birth group [median (interquartile range), 1.00*10^8^(7.59*10^7^- 1.39*10^8^) *vs* 1.01*10^8^ (7.37*10^7^- 1.32*10^8^)]. After controlling for potential confounders in a logistic regression analysis with generalized estimating equations, the relationship between the mtDNA content and live birth remained non-significant (OR=1.000, 95% CI 1.000-1.000, p=0.567), as shown in **Table 3**. Further, to determine whether the mtDNA content in day-5 or day-6 embryos was related to embryo viability, we compared the mtDNA content between different transfer outcomes in day-5 and day-6 embryos respectively (**Table 3**). Similarly, no association between mtDNA content and live birth was observed (p=0.673, p=0.165, respectively).

**Table 2:** The epidemiological and clinical characteristics of the all patients undergoing IVF/PGT treatment.

Characteristic	Live birth N=105	Miscarriage N=26	Not-implanted N=185	p value
**Maternal age, y, mean ± SD**	32.3 ± 4.7	31.6 ± 4.7	33.2 ± 4.7	0.116
**Paternal age, y, mean ± SD**	34.6 ± 6.1	34.0 ± 4.7	34.5 ± 5.2	0.896
**BMI, kg/m2, mean ± SD**	21.5 ± 2.5	21.0 ± 1.9	21.6 ± 2.4	0.315
**Education, n (%)**				0.903
Senior high school degree or less	14 (28.6)	5 (10.2)	30 (61.2)	
college degree	78 (33.6)	19 (8.2)	135 (58.2)	
post-graduate degree	13 (37.1)	2 (5.7)	20 (57.1)	
gravidity	1 (0-2)	1 (0-3)	1 (0-2)	0.512
parity	0 (0-0)	0 (0-0)	0 (0-0)	0.554
**Cycle protocols, n (%)**				0.693
GnRH agonist protocol	40 (32.8)	11 (9.0)	71 (58.2)	
Antagonist protocol	57 (34.1)	13 (7.8)	97 (58.1)	
microdose flare protocol	8 (36.4)	1 (4.5)	13 (59.1)	
Other protocol	0 (0.0)	1 (20.0)	4 (80.0)	
**Indications for PGT, n (%)**				0.538
chromosomal abnormalities	32 (29.6)	12 (11.1)	64 (59.3)	
Advanced age	14 (32.6)	2 (4.7)	27 (62.8)	
Male factor	17 (40.5)	2 (4.8)	23 (54.8)	
Recurrent implantation failure	4 (28.6)	2 (14.3)	8 (57.1)	
Recurrent miscarriage	15 (34.9)	6 (14.0)	22 (51.2)	
Patient request	23 (34.8)	2 (3.0)	41 (62.1)	
**Infertility type, n (%)**				0.694
Primary	38 (36.2)	9 (8.6)	58 (55.2)	
Secondary	67 (31.8)	17 (8.1)	127 (60.2)	
**bFSH, IU/L**	7.2 ± 2.0	6.8 ± 2.7	7.3 ± 2.4	0.157
**bLH, IU/L**	4.8 ± 2.2	6.1 ± 3.2	4.5 ± 2.1	0.082
**bEstradiol, pmol/L**	141.7 ± 75.8	155.7 ± 75.0	140.5 ± 66.0	0.712
**Gonadotropin duration, days**	10.0 ± 2.7	9.7 ± 1.9	9.8 ± 2.3	0.900
**Gonadotropin dosage, IU**	2302.7 ± 778.9	2422.6 ± 449.3	2359.0 ± 738.0	0.599
**endometrial thickness, mm**	9.3 ± 1.4	9.7 ± 2.0	9.4 ± 1.5	0.717
**FET type, n (%)**				0.617
Natural cycle	35 (32.1)	7 (6.4)	67 (61.5)	
HRT cycle	70 (33.8)	19 (9.2)	118 (57.0)	
**Biopsy day, n (%)**				0.004*
5	35 (49.3)	3 (4.2)	33 (46.5)	
6	70 (28.6)	23 (9.4)	152 (62.0)	
**TE quality, n (%)**				0.099
A	6 (60.0)	0 (0)	4 (40.0)	
B	75 (36.2)	16 (7.7)	116 (56.0)	
C	24 (24.2)	10 (10.1)	65 (65.7)	
**ICM quality, n (%)**				0.070
A	14 (38.9)	6 (16.7)	16 (44.4)	
B	91 (32.5)	20 (7.1)	169 (60.4)	
C	0 (0)	0 (0)	0 (0)	
**Blastocyst quality, n (%)**				0.024*
Good	14 (38.9)	6 (16.7)	16 (44.4)	
Average	67 (37.0)	10 (5.5)	104 (57.5)	
Poor	24 (24.2)	10 (10.1)	65 (65.7)	

Continuous variables were calculated by Kruskal-Wallis test, categorical variables were calculated by chi-squared test; BMI, body mass index; PGT, preimplantation genetic testing; TE, Trophectoderm; ICM, inner cell mass; bLH, basic luteinizing hormone; bFSH, basic follicle-stimulating hormone; bAMH, basic anti-Müllerian hormone; FET, frozen-thawed embryo transfer; HRT, hormone replacement therapy; *p<0.05.

**Table 3:** Results of Logistic regression with generalized estimating equations on IVF-PGT outcomes (live birth, miscarriage, non-implanted).

Variables	All blastocysts		D5 blastocysts		D6 blastocysts	
	aOR^a^ (95% CI)	P	aOR^b^ (95% CI)	P	aOR^b^ (95% CI)	P
mtDNA content	1.000 (1.000-1.000)	0.567	1.000 (1.000-1.000)	0.673	1.000 (1.000-1.000)	0.165
Maternal age, years	1.033 (0.983-1.086)	0.197	1.105 (0.998-1.225)	0.055	1.026 (0.967-1.087)	0.397
bLH, IU/L	0.987 (0.895-1.089)	0.798	1.091 (0.926-1.285)	0.299	0.974 (0.865-1.096)	0.661
Blastocyst quality						
Good	0.639 (0.213-1.920)	0.425	0.849 (0.145-4.967)	0.856	0.530 (0.251-1.122)	0.097
Average	0.662 (0.198-2.209)	0.502	1.298 (0.386-4.366)	0.674	0.533 (0.317-0.895)	0.017
Poor	reference		reference		reference	
Biopsy day						
5	0.730 (0.246-2.161)	0.569				
6	reference					

a adjusted for maternal age, basic luteinizing hormone, biopsy day and blastocyst quality.

b adjusted for maternal age, basic luteinizing hormone and blastocyst quality.

aOR, adjusted odds ratio; CI, confidence interval; bLH, basic luteinizing hormone.

**Figure 3:**
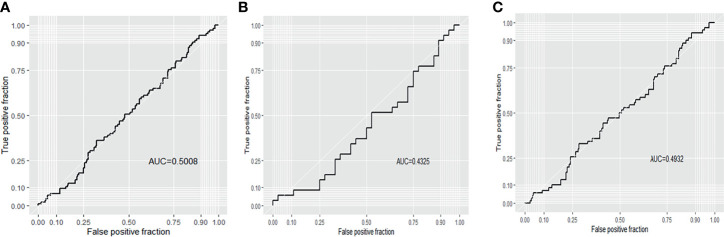
Receiver operating characteristic (ROC) curve analysis of live birth for the 316 transferred blastocysts. **(A)** all blastocysts; **(B)** D5 blastocysts; **(C)** D6 blastocysts.

According to receiver operating characteristic (ROC) curve analysis, the area under curve (AUC) was 0.501 (94.3% sensitivity, 10.9% specificity) for all blastocysts, 0.433 (91.4% sensitivity, 25.0% specificity) for Day 5 biopsied blastocysts and 0.493 (90.0% sensitivity, 18.9% specificity) for Day 6 biopsied blastocysts, which indicates that mtDNA content has a poor predictive value for live birth (**Figure 3**).

**Figure 4:**
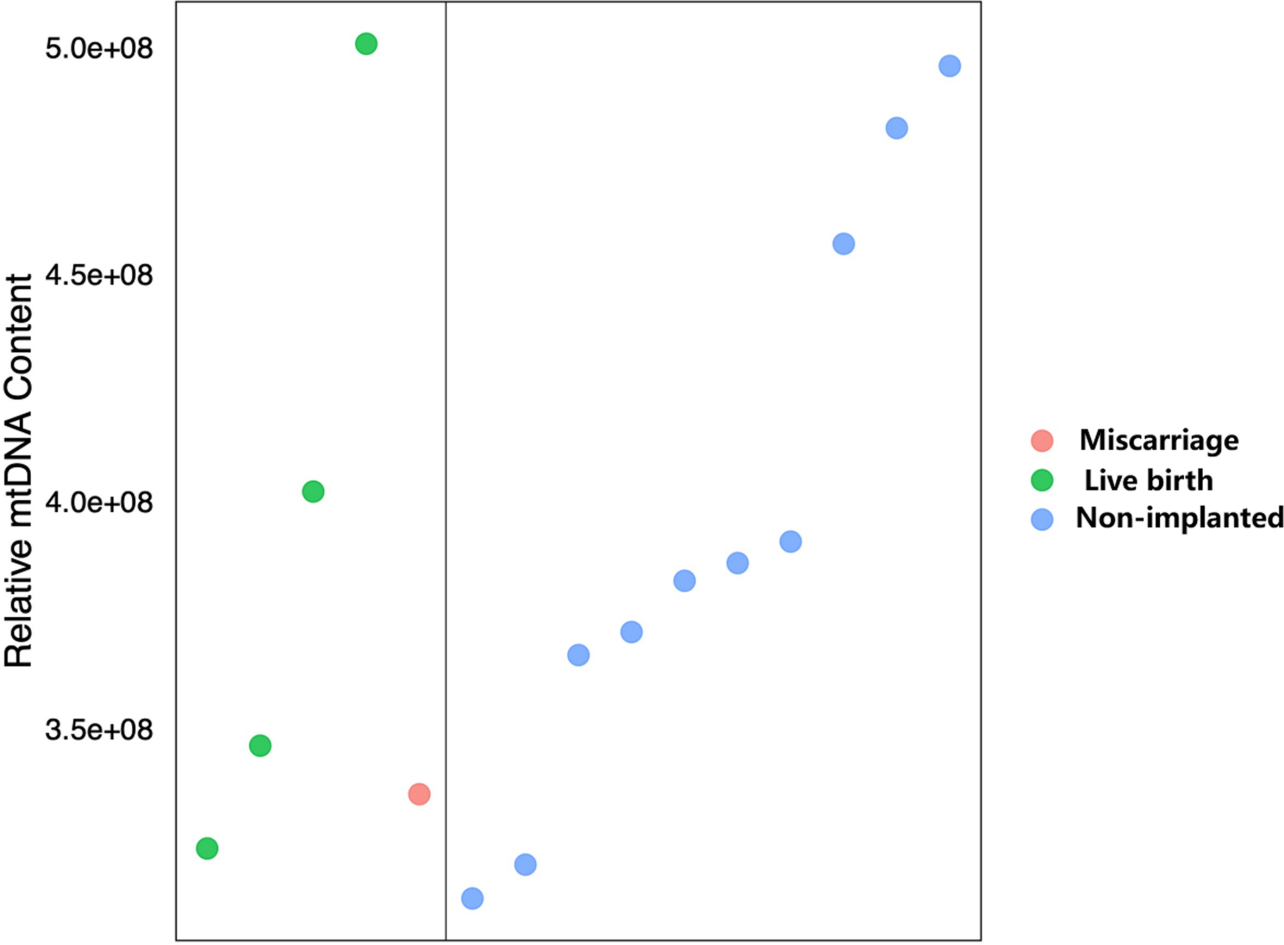
Transfer outcomes of blastocysts with an elevated mtDNA content.

To further evaluate the predictive value of an elevated mtDNA level in reproductive competence, blastocysts with an elevated mtDNA content (above the 95th percentile) were further analysed. Of these 15 blastocysts, four reached live birth, one resulted in miscarriage and the remaining ten blastocysts resulted in non-implanted (**Figure 4**). The live birth rate among these blastocysts were not significantly different from those among blastocysts with a normal mtDNA content (26.7% *vs* 33.6%, p=0.780). In addition, it is worth mentioning that the blastocyst containing the highest mtDNA content was from a 44-year-old woman, and the blastocyst resulted in a successful live birth, which suggests that embryos with a higher mtDNA content also have developmental potential for successful pregnancy and live birth.

**Figure 5:**
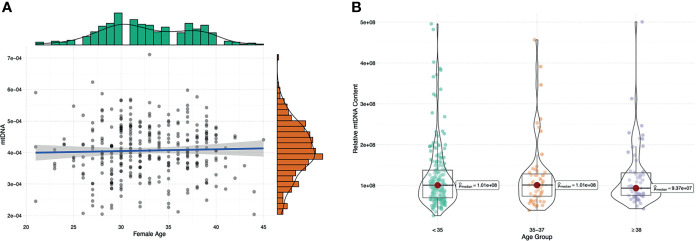
The relationship between the mtDNA content and maternal age. **(A)** Linear regression analysis, blue lines indicate linear regressions; gray shading indicates 95% confidence regions for linear regressions. **(B)** subgroup analysis of different maternal ages (<35, 35-37 and ≥38 years); Kruskal-Wallis test was used to compare the mtDNA content across different age groups and p values less than 0.05 were considered significant.

**Figure 6:**
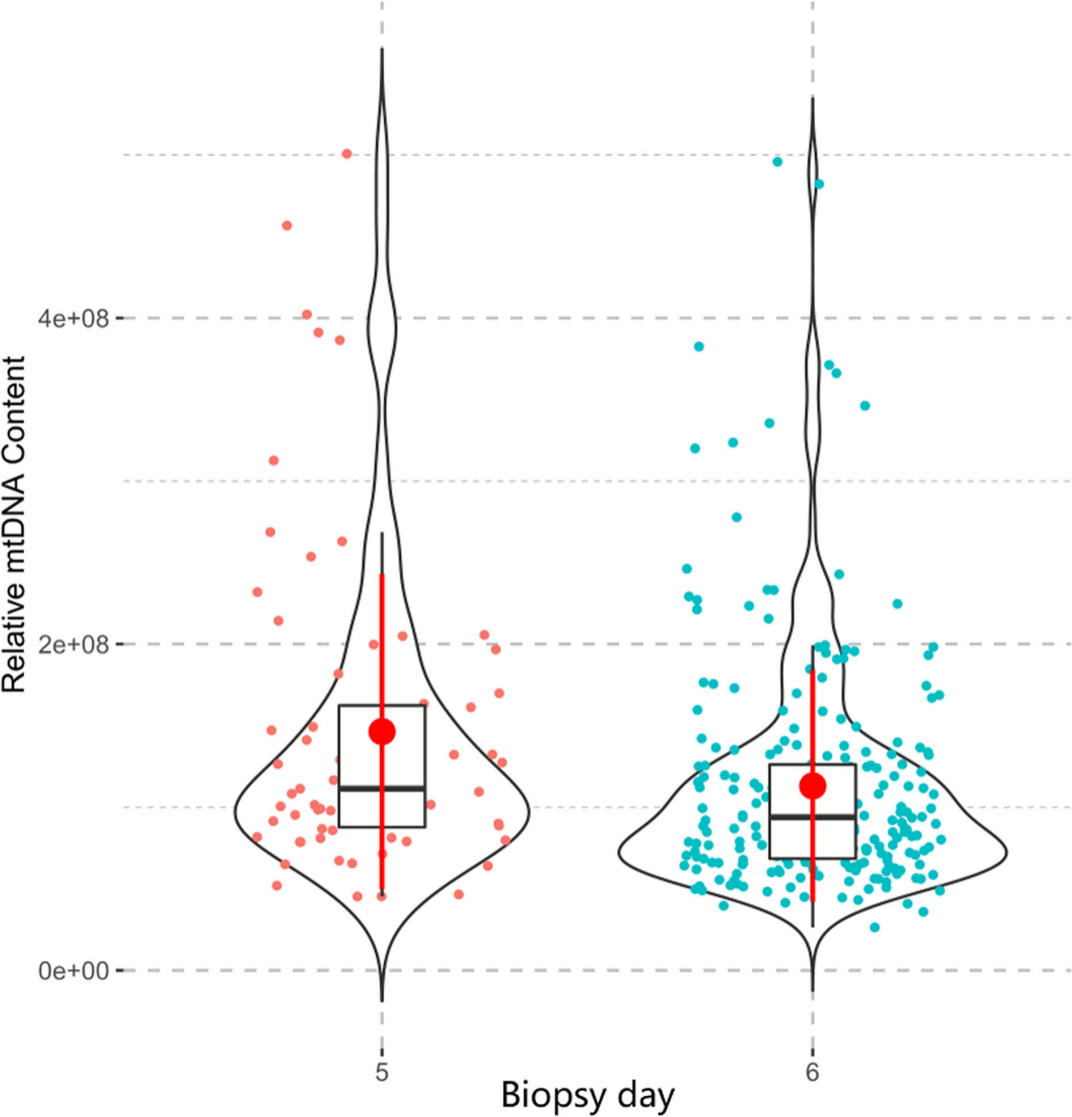
The relationship between the mtDNA content and biopsy day. The Mann-Whitney test was performed to compare the mtDNA content between two groups, Day-5 blastocysts showed higher level of mtDNA content than Day-6 blastocysts (p=0.001).

Maternal age and biopsy day were evaluated for associations with mtDNA content in all 316 embryos. There was no linear correlation between the mtDNA content and maternal age (p=0.570, **Figure 5A**) in this study. Further age subgroup analysis also showed no significant difference in the mtDNA content (p=0.997, **Figure 5B**) among different age groups (<35, 35-37and ≥38 years). Blastocysts biopsied on Day 5 showed higher levels of mtDNA content compared with those biopsied on Day 6 (p=0.001, **Figure 6**).

## Discussion

Currently, morphological assessment is the primary and most common method for determining which embryo is more suitable for transfer and may yield superior outcomes (52). However, the description of morphological characteristics is subjective, which makes it harder to achieve complete standardization in evaluating the viability and developmental competence of embryos (53, 54). The content of mtDNA, calculated by the ratio of mitochondrial DNA: nuclear DNA (34), is a good objective indicator that has recently been proposed as a new biomarker of embryo viability. Fragouli et al. and Diez-Juan et al. first demonstrated that increased mtDNA levels in embryos were related to poor implantation potential in 2015 (27, 28). Since then, quite a few scholars have conducted research into the relationship of the mtDNA content with embryo viability and transfer outcomes (29–33. 34, 55). Several studies have arrived at the same conclusion (29, 30, 55), whereas others have not (31–33). Victor et al. showed no significant difference in the mtDNA levels between implanted and nonimplanted blastocysts regardless of whether the samples were detected by NGS or qPCR (34). The author also recommended the use of correction factors to normalize the mtDNA content determination among centers, which may reconcile the discrepant conclusions of existing studies. In this study, the calculation of the mtDNA content was corrected by the mathematical factor proposed by Victor et al. (34), and we failed to find any association between the mtDNA content and embryo transfer outcomes (i.e., implantation and live birth). These findings are consistent with those of other studies that also used the same correction factor (35, 56). However, Wang et al. determined the mtDNA content using an NGS-based methodology with mathematical correction but reached the opposite conclusion (57). Wang et al. found that the mtDNA content was significantly associated with embryo implantation and live birth, with lower mtDNA levels leading to higher implantation and live birth rates. One possible explanation is that the range of mtDNA content in human blastocysts varies among centers. In a relatively large, multicenter study by Ravichandran et al. (29), 282 euploid blastocysts from 35 different IVF centers were analysed. The authors found that only half of the centers detected blastocysts with an elevated mtDNA level, and the proportion of blastocysts with a high mtDNA levels did not exceed 27%. Another prospective study from a single large IVF centre showed that 9/199 (4.5%) blastocysts contained high levels of mtDNA (30). In fact, blastocysts with a high mtDNA content seem to be relatively rare, and elevated levels of mtDNA could be associated with culture conditions during laboratory procedures. In addition, the methods used for mtDNA quantification in different studies were not all the same. Thus, the threshold for elevated levels of mtDNA, however, might not be fixed and should be adjusted according to the methods used for mtDNA calculation and culture conditions for embryos in different IVF centers. This may account for some discrepancies among the existing reports. Therefore, the mtDNA content detected in this study, even the highest mtDNA content, may still be in the “normal range” of a euploid blastocyst. It is also understandable that the blastocyst with the highest mtDNA content can achieve a live birth at last.

Mitochondria are the main source of energy in cells, providing more than 95% of the ATP required for cell metabolism (58). The maintenance of a healthy mitochondrial network depends on the proper regulation of mitochondrial quantity and quality. The mitochondrial content is biologically modulated to accelerate ATP generation and facilitate changes in respiratory capacity to keep up with cellular metabolic demands (59, 60). It has been suggested that mtDNA activity and content increase when embryos are under oxidative stress (61, 62). Thus, an elevated mitochondrial content may be a marker of oxidative and metabolic stress in embryos but not a direct marker of mitochondrial dysfunction. Thus, embryos with a higher mtDNA content might also have the potential to develop into a fetus after implantation. Furthermore, according to the quiet embryo hypothesis and Goldilock’s principle (63, 64), the levels of mtDNA in embryos with a better potential for development will be located in a suitable range, called Goldilock’s zone. Embryos lacking sufficient energy reserves usually show a compensatory increase in the mtDNA content to meet the energy requirement during embryo development. Therefore, for embryos with a mild energy deficiency, such a remedy could be very useful and the mtDNA content may still in the Goldilock’s range, the embryo also has the potential for implantation and live birth. Once an embryo is implanted, a higher mtDNA content is less likely to result in adverse outcomes, which is supported by the finding in our study that the mtDNA content was similar in blastocysts resulting in miscarriage and live birth.

Furthermore, endometrial receptivity, embryo viability, and maternal–fetal tolerance are three important determinants of successful embryo implantation (65). Few prior studies have considered the potential confounding effects of maternal factors. In the study, women with autoimmune and endometrial diseases were excluded from this study, and we still found that no relationship observed between different embryo transfer outcomes. Thus, the mtDNA content seems unable to predict embryo implantation or live birth.

Discrepant results have been reported concerning the association of mtDNA with maternal age. Klimczak et al. and Lee et al. reported that the mtDNA content had no correlation with maternal age (33, 35). We failed to find a linear correlation between mtDNA and maternal age, which is consistent with previous studies. After the sub-group analysis of different maternal age groups. Fragouli et al. found a higher mtDNA content in women aged ≥38 years than in those aged <38 years (27), while Treff et al. obtained the opposite results that mtDNA content decreased with advancing age (31). Other studies found no relation with maternal age (28, 34, 57), which is consistent with our finding. Although the hypothesis that reproductive ageing in older women is associated with the content of mtDNA in blastocyst-stage embryos is attractive (27), our results do not support this argument. We also found that blastocysts biopsied on Day 5 showed higher levels of mtDNA content compared with those biopsied on Day 6, which was consistent with previous studies (33, 55, 57, 66). The replication of mtDNA will not be reactivated in the embryo until the blastocyst stage (67), blastocysts biopsied on Day 6 may undergo more cell divisions compared with blastocysts biopsied on Day 5, which may result in a reduction of mtDNA content in Day 6 blastocysts. However, one study by Wu et al. suggest another explanation (66), they proposed a hypothesis claiming that Day 6 blastocysts are developed from those oocytes which contain lower levels of mtDNA content. This means more time and efforts would be required for these embryos to develop into the blastocyst stage. Therefore, the levels of mtDNA content in Day 6 blastocysts are relatively low.

Although the blastocysts biopsied on Day 5 showed a higher potential for implantation and live birth, and Day 5 blastocysts contained higher levels of mtDNA content compared with Day 6 blastocysts. However, this does not imply that elevated mtDNA content is related to implantation or live birth. The level of mtDNA content is just one of the potential factors which could affect the transfer outcomes of Day 5 blastocysts. Other factors, such as embryo quality, may also have an impact on transfer outcomes. Given that the results of logistic regression and ROC curve analysis, we still conservatively considered that mtDNA content is not a reliable screening biomarker for live birth after embryo transfer.

Despite careful selection of the patients, there are some limitations in the present study. First, the sample size for this epidemiological study was relatively small, future studies need to expand the sample size for further generalization. Second, the biopsy samples were collected at a single IVF centre, which may influence the generalizability of our findings despite the consistency of the culture conditions and testing methodologies.

Taken together, the findings of this study indicate that the mtDNA content has poor value for predicting embryo transfer outcomes (i.e., implantation and live birth). The mtDNA content did not differ between blastocysts resulting in miscarriage and live birth. Additionally, the mtDNA content was not correlated with maternal age. We recommend that more stringent inclusion and exclusion criteria and mtDNA quantification standards should be taken into account in subsequent studies.

## Data Availability Statement

The data presented in the study are deposited in the SRA repository, accession number PRJNA764530.

## Ethics Statement

The studies involving human participants were reviewed and approved by the ethics committee of the International Peace Maternal and Child Health Hospital. The patients/participants provided their written informed consent to participate in this study.

## Author Contributions

Conceived and designed the experiments: XZ and SC. Performed the experiments: XL, MY, and WS Analyzed the data: WS. Wrote the paper: XZ. All authors contributed to the article and approved the submitted version.

## Funding

This work was supported by the National Natural Science Foundation of China (Nos.81971344, 81771638 and 81901495), the research grant from the National Key R&D Program of China (2018YFC1004900) the Shanghai Municipal Commission of Science and Technology Program (No.21Y21901002) Shanghai Municipal Health Commission (GW-10.1-XK07), the Shanghai “Rising Stars of Medical Talent” Youth Development Program Clinical Laboratory Practitioners Program (201972) Shanghai Municipal Commission of Health and family planning (202140110, 20215Y0216).

## Conflict of Interest

The authors declare that the research was conducted in the absence of any commercial or financial relationships that could be construed as a potential conflict of interest.

## Publisher’s Note

All claims expressed in this article are solely those of the authors and do not necessarily represent those of their affiliated organizations, or those of the publisher, the editors and the reviewers. Any product that may be evaluated in this article, or claim that may be made by its manufacturer, is not guaranteed or endorsed by the publisher.
