# Supplementary material for: Mitochondrial DNA Content May Not Be a Reliable Screening Biomarker for Live Birth After Single Euploid Blastocyst Transfer
Source: Front Endocrinol (Lausanne). 2021 Nov 16;12:762976. doi: 10.3389/fendo.2021.762976 (PMC8637898; doi:10.3389/fendo.2021.762976)
Supplement: Supplementary file 1 [file Table_1.docx]

Supplementary Table 1 Results of Logistic regression with generalized estimating equations on IVF-PGT outcomes (implanted and non-implanted)

| Variables | All blastocysts | | D5 blastocysts | | D6 blastocysts | |
| --- | --- | --- | --- | --- | --- | --- |
|  | aOR^a^（95% CI） | P | aOR^b^（95% CI） | P | aOR^b^（95% CI） | P |
| mtDNA content | 1.000(1.000-1.000) | 0.207 | 1.000(1.000-1.000) | 0.734 | 1.000(1.000-1.000) | 0.394 |
| Maternal age, years | 0.951(0.902-1.003) | 0.066 | 0.903(0.811-1.006) | 0.064 | 0.963(0.904-1.025) | 0.239 |
| bLH, IU/L | 1.081(0.968-1.206) | 0.167 | 0.944(0.789-1.129) | 0.526 | 1.082(0.950-1.232) | 0.237 |
| Blastocyst quality |  |  |  |  |  |  |
| Good | 2.192(0.984-4.884) | 0.055 | 1.205(0.185-7.845) | 0.845 | 2.151(0.948-4.880) | 0.067 |
| Average | 1.314(0.816-2.118) | 0.261 | 0.513(0.136-1.937) | 0.325 | 1.535(0.955-2.468) | 0.077 |
| Poor | reference |  | reference |  | reference |  |
| Biopsy day |  |  |  |  |  |  |
| 5 | 1.830(1.034-3.239) | 0.038 |  |  |  |  |
| 6 | reference |  |  |  |  |  |

^a^ adjusted for maternal age, basic luteinizing hormone, biopsy day and blastocyst quality;

^b^ adjusted for maternal age, basic luteinizing hormone and blastocyst quality;

aOR, adjusted odds ratio; CI, confidence interval; bLH, basic luteinizing hormone;
